# Characterisation of cytotoxicity and immunomodulatory effects of glycolipid biosurfactants on human keratinocytes

**DOI:** 10.1007/s00253-022-12302-5

**Published:** 2022-11-28

**Authors:** Simms A. Adu, Matthew S. Twigg, Patrick J. Naughton, Roger Marchant, Ibrahim M. Banat

**Affiliations:** School of Biomedical Science, Ulster University, Coleraine, BT52 1SA UK

**Keywords:** Glycolipid biosurfactants, Sodium lauryl ether sulphate, Keratinocytes, Cosmetics and personal care, Immunomodulation, Skin irritation, Skincare formulations

## Abstract

**Abstract:**

Skin irritation and allergic reactions associated with the use of skincare products formulated with synthetically derived surfactants such as sodium lauryl ether sulphate (SLES) have encouraged the search for naturally derived and biocompatible alternatives. Glycolipid biosurfactants such as sophorolipids (SL) and rhamnolipids (RL) offer a potential alternative to SLES. However, most studies on the bioactive properties of microbial glycolipids were determined using their mixed congeners, resulting in significant inter-study variations. This study aims to compare the effects of highly purified SL (acidic and lactonic) and RL (mono-RL and di-RL) congeners and SLES on a spontaneously transformed human keratinocyte cell line (HaCaT cells) to assess glycolipids’ safety for potential skincare applications. Preparations of acidic SL congeners were 100% pure, lactonic SL were 100% pure, mono-RL were 96% pure, and di-RL were 97% pure. Cell viability using XTT assays, cell morphological analyses, and immunoassays revealed that microbial glycolipids have differing effects on HaCaT cells dependent on chemical structure. Compared with SLES, acidic SL and mono-RL have negligible effects on cell viability, cell morphology, and production of pro-inflammatory cytokines. Furthermore, at non-inhibitory concentrations, di-RL significantly attenuated IL-8 production and *CXCL8* expression while increasing IL-1RA production and *IL1RN* expression in lipopolysaccharide-stimulated HaCaT cells. Although further studies would be required, these results demonstrate that as potential innocuous and bioactive compounds, microbial glycolipids could provide a substitute to synthetic surfactants in skincare formulations and perform immunopharmacological roles in topical skin infections such as psoriasis.

**Key points:**

*• Purified glycolipid congeners have differing effects on human keratinocytes.*

*• Compared with SLES, acidic sophorolipids and mono-rhamnolipids have innocuous effects on keratinocytes.*

*• Di-rhamnolipids and mono-rhamnolipids modulate cytokine production in lipopolysaccharide stimulated human keratinocytes.*

**Supplementary Information:**

The online version contains supplementary material available at 10.1007/s00253-022-12302-5.

## Introduction

Cosmetics and personal care products are formulated to function as an added nutritional source to the human skin, improve skin barrier functions, inhibit the growth of pathogenic microorganisms, cleanse, and moisturise skin surfaces (Heinrich et al. 2014; Rodan et al. 2016; Purnamawati et al. 2017; Yamaguchi et al. 2017; Bouslimani et al. 2019). Despite these health benefits and the subsequent ubiquitous and frequent use of cosmetics and personal skincare products, many of the component ingredients used in their base formulations are often synthesised from petrochemical resources; a key example are surfactants such as synthetic sodium lauryl ether sulphate (SLES), which can make up to 50% (v/v) of the formulation and play a role in emulsification, gelling, and micro-encapsulation (Leoty-Okombi et al. 2021; Moldes et al. 2021).These synthetically derived surfactants have drawbacks with regards to their sustainability and are less biodegradable than biologically derived alternatives (Marchant and Banat 2012; Suhail et al. 2019; Goyal and Jerold 2021). Synthetically derived surfactants such as SLES are also reported to have the potential to cause allergic reactions, skin irritations, and dysbiosis in the skin microbiome when they come into direct contact with the human skin (Bouslimani et al. 2019; Mijaljica et al. 2022). In such instances, SLES binds to lipids and proteins on the epidermal layer of the human skin resulting in their solubilisation, the production of cytokines, chemokines, and other pro-inflammatory mediators. This ultimately destabilises the structural integrity of the skin and subsequently results in transepidermal water loss (Seweryn 2018). As such, there is a current market demand to replace synthetic ingredients in cosmetics and personal skincare formulations with naturally derived and biocompatible alternatives generated from sustainable resources (Otzen 2017; Mohiuddin 2019; Goyal and Jerold 2021).

Biosurfactants are naturally derived surfactants produced as secondary metabolites by bacteria, yeast, and filamentous fungi (Banat et al. 2010; Naughton et al. 2019; Da Silva et al. 2021; Manga et al. 2021). The classification of microbial biosurfactants is based on their chemical structure, molecular weight, and microbial origin (Ceresa et al. 2021; Moldes et al. 2021; Sarubbo et al. 2022). Glycolipids comprised of a carbohydrate moiety linked to long-chain aliphatic acids or hydroxy aliphatic acids of varying lengths, which constitute the most extensively studied and biotechnologically promising class of biosurfactants (Bhattacharya et al. 2017; Shu et al. 2021). Glycolipids can be further classified into rhamnolipids, sophorolipids, trehalolipids, and mannosylerythritol lipids (Thakur et al. 2021). Among these, rhamnolipids and sophorolipids are the most abundant, extensively studied, and promising groups. Rhamnolipids are produced by gram-negative bacteria such as *Pseudomonas*, *Burkholderia*, and *Marinobacter* species; they consist of one (mono-rhamnolipids) or two rhamnose (di-rhamnolipids) as the hydrophilic moiety bonded to a hydrophobic moiety of one or two β-hydroxy fatty acid chains of 8–16 carbons (Funston et al. 2016; Twigg et al. 2018; Tripathi et al. 2019). Sophorolipids, produced by yeast species such as *Starmerella bombicola*, comprise of a hydrophilic head (sophorose) bonded to either esterified (lactonic sophorolipids) or non-esterified (acidic sophorolipids) hydroxy fatty acid tail lengths of 16–18 carbons (Santos et al. 2016). The potential advantages of utilising glycolipids over synthetic surfactants such as SLES in cosmetics and personal skincare formulation are low toxicity, biodegradability, and increased compatibility with the human skin (Fracchia et al. 2015; Naughton et al. 2019; Fenibo et al. 2019; Adu et al. 2020). For skincare applications, the safety of glycolipids is of particular importance for incorporation into skincare formulations. Hence, the safety of glycolipids in skincare formulations is usually ascertained in vitro by assessing their cytotoxicity effects on various mammalian skin cell types (Inès and Dhouha 2015; Maeng et al. 2018; Moldes et al. 2021).

The cytotoxicity effects of rhamnolipids and sophorolipids have been demonstrated in vitro against mouse skin fibroblasts (NCTC clone 929), spontaneously transformed human keratinocyte cell line (HaCaT cells), and normal human dermal fibroblastic cells (Lydon et al. 2017; Maeng et al. 2018; Haque et al. 2020; Rodríguez-López et al. 2020; Voulgaridou et al. 2021). However, most of these studies utilised either impure preparations, poorly characterised or single class of glycolipids resulting in significant interstudy variations, which in effect render glycolipids less attractive for use in skincare applications. Moreover, most in vitro studies on glycolipids only focused on their effects on cell viability rather than comprehensive studies involving the investigation of potential glycolipid mechanisms of cell death induction and the production/modulation of cytokines (Callaghan et al. 2016; Lydon et al. 2017). Therefore, to broaden the potential applications of glycolipids and to make them more attractive for skincare applications, this study aims to comprehensively assess the cytotoxicity and immunomodulatory effects of purified and fully characterised glycolipid congeners on HaCaT cells and compare with SLES. We, therefore, hypothesised that in comparison with SLES, the purified glycolipid congeners utilised in this study will have not deleterious effects on human keratinocytes, but will provide added functionality to skin cells.

Using a combination of in vitro cell culture, molecular biology techniques, and immune assays, we have demonstrated that the purified microbial glycolipid congeners have differing effects on human keratinocytes depending on their chemical structure. Moreover, compared with SLES, some glycolipid congeners demonstrated negligible effects on cell viability, cell morphology, the production of pro-inflammatory cytokines, and the expression of their related genes. Furthermore, these glycolipids attenuated pro-inflammatory cytokine production following stimulation with pathogen-associated molecular patterns (PAMPSs). These findings suggest that as potential innocuous and naturally derived surfactants, microbial glycolipids could potentially offer a safer and suitable alternative to SLES in skincare formulations and, as an added functionality, perform immunopharmacological roles in topical skin infections such as psoriasis.

## Materials and methods

### Purification, chemical characterisation, and analysis of glycolipids’ surface activity

Purified non-acetylated acidic sophorolipids (acidic SL) and di-acetylated lactonic sophorolipids (lactonic SL) were obtained from *Biosynth Carbosynth*, Compton, UK. Rhamnolipids were obtained from *Daqing Victex Chemical Co. Ltd.*, Daqing, China, as crude mixture of mono-rhamnolipid (mono-RL) and di-rhamnolipid (di-RL) congeners and purified *in-house* via liquid and solid-phase extractions. All glycolipid congeners were chemically characterised using high-performance liquid chromatograph-mass spectrometry coupled electrospray ionisation mass spectrometer (HPLC–MS/ESI) as described in previous work (Adu et al. 2022). Stock preparations of each glycolipid congener were prepared at a concentration of 1 mg mL^−1^ in 1% (v/v) HPLC-grade methanol (*Merck*, Gillingham, UK) and stored at − 20 °C. For CMC determination, the stock preparations of each glycolipid congener were further diluted in sterile distilled water to a concentration gradient of 0.04–1 mg mL^−1^. SLES (*R & D Laboratories Limited*, Antrim, UK) was diluted in sterile distilled water to a concentration gradient of 0–5.27 mg mL^−1^. CMC was determined using Krüss K10 ST digital tensiometer (Krüss K10 ST, Hamburg, Germany) via the Du Noüy platinum ring method previously described by Rodríguez-López et al. (2020). Surfactant concentration against surface tension was plotted, and the CMC was determined from extrapolated intercepts of the *X* and *Y* axes (Rodríguez-López et al. 2020).

### Cell culture

A spontaneously transformed human keratinocyte (HaCaT cells) (T0020001/117) cell line utilised in this study was sourced from *AddexBio*, San Diego, CA, USA. HaCaT cells were routinely cultured in high-glucose DMEM (*ThermoFisher Scientific*, Loughborough, UK) supplemented with 10% (v/v) foetal bovine serum (FBS) (*ThermoFisher Scientific*, Loughborough, UK) and 1% (v/v) sodium pyruvate (*ThermoFisher Scientific*, Loughborough, UK). Cells were cultured at 37 °C in a humidified atmosphere containing 5% CO_2_.

### Cell viability assays

The viability of HaCaT cells treated with varying concentrations of each glycolipid congener and SLES was assessed using a cell proliferation assay II (XTT) kit (*Roche*, Welwyn Garden City, UK). HaCaT cells were cultured to confluency, seeded into 96-well cell culture plates (*Sarstedt*, Leicester, UK) at a density of 1 × 10^4^ cells per well, and cultured for 24 h. Cells were serum-starved for 24 h and then treated with media supplemented with either 1% (v/v) HPLC-grade methanol (vehicle control) (*Merck*, Gillingham, UK) or with incremental concentrations of each surfactant preparation (0–100 μg mL^−1^) for a further 24 h. For acidic SL and mono-RL congeners, a further experiment treating the cells with an increased concentration up to 500 μg mL^−1^ was performed. Following treatment, the medium was aspirated, and the cells were washed three times with sterile phosphate-buffered saline (PBS) (*ThermoFisher Scientific*, Loughborough, UK). Pre-prepared XTT medium (*Roche*, Welwyn Garden City, UK) was added to the cells (50 μL per well) and incubated for 4 h. Post incubation, absorbance was measured at 450 and 650 nm using a FLUOstar Omega microplate reader (*BMG Labtech*, Offenburg, Germany). Viability of HaCaT cells post treatment with either glycolipids or SLES was expressed as a percentage relative to the untreated control group. Furthermore, lethal dose 50% (LD_50_) values were determined by nonlinear regression curves using Prism v 9.4.1 (458) for MacOS (*GraphPad Software*, San Diego, CA, USA).

### Cell morphology assessment

HaCaT cellular morphology following treatment with each glycolipid congener or SLES was assessed by directly observing the cells using visible light microscopy. HaCaT cells were grown to confluency, seeded into 12-well cell culture plates (*Sarstedt*, Leicester, UK) at a density of 1 × 10^5^ cells per well, and cultured for 24 h. Cells were serum-starved for 24 h and treated for a further 24 h in complete medium supplemented with either 1% (v/v) HPLC-grade methanol (V. ctrl) (*Merck*, Gillingham, UK) or 20 μg mL^−1^ and 100 μg mL^−1^ of each surfactant. These treatment concentrations were chosen to assess the effects on morphology of the HaCaT cells induced by the surfactants at the lowest and highest concentrations utilised in the present study. As with the cell viability experiments, a further experiment with acidic SL and mono-RL congeners at an increased treatment concentration of up to 500 μg mL^−1^ was also carried out. Following treatment, the morphology of HaCaT cell was assessed by directly imaging the cells in the wells at 200 × magnification using a Digital Sight DS-L1 camera (*Nikon Europe B. V.*, Amsterdam, The Netherlands) attached to an Eclipse TS100 inverted microscopy (*Nikon Europe B. V.*, Amsterdam, The Netherlands).

### Acridine orange (AO) and propidium iodide (PI) staining

To determine the distinct morphological pattern of HaCaT cell death induced following treatment with each glycolipid congener and SLES, treated cells were stained with AO and PI (Lee et al. 2015). Experiments were set up and cells treated as described in the previous sub-section “Cell morphology assessment”. Following treatment, the cells were washed three times with sterile PBS (*ThermoFisher Scientific*, Loughborough, UK) to remove detached cells and subsequently incubated with a 1:1 ratio of 100 μg mL^−1^ AO and PI (*Merck*, Gillingham, UK) for 3 min. To remove excess stains, the cells were washed three times with prewarmed sterile PBS (*ThermoFisher Scientific*, Loughborough, UK) and the stained cells were immediately imaged at 200 × magnification using Eclipse TS100 fluorescence microscope (*Nikon Europe B. V.*, Amsterdam, The Netherlands). The excitation and emission wavelengths for AO were 493 and 535 nm, and for PI, 535 and 614 nm, respectively.

### Assessment of pro-inflammatory cytokine production

Both a semi-quantitative array and enzyme-linked immunosorbent assays (ELISA) for individually selected cytokines were used to investigate the effect of surfactant treatment on the production of pro-inflammatory cytokines in HaCaT cells. Supernatant samples generated from treated HaCaT cells were initially assayed using a semi-quantitative multiplexed Proteome Profiler Human Cytokine Array Kit (*R&D Systems, Inc.,* MN, USA). HaCaT cells were grown to confluency and seeded into 6-well cell culture plates (*Sarstedt*, Leicester, UK) at a density of 3 × 10^5^ cells per well for 24 h. Cells were then serum starved for 24 h and treated with complete medium supplemented with either 1% (v/v) HPLC-grade methanol (V. ctrl) (*Merck*, Gillingham, UK); 25 μg mL^−1^ of lipopolysaccharide (LPS) from *Escherichia coli* (*Merck*, Gillingham, UK) (positive control for the assay); or LD_50_ concentrations of lactonic SL and di-RL (63 μg mL^−1^ and 48 μg mL^−1^, respectively). Treated cultures were centrifuged at 1000 × g to generate cell-free supernatant samples which were incubated on nitrocellulose membranes as per the manufacturer’s instructions. Dot blots developed on nitrocellulose membranes were imaged using a G: BOX Chemi XRQ (*Syngene*, Cambridge, UK), and the densitometry of each dot was analysed using ImageJ Software (Schneider et al. 2012) .

Following the initial profiling of pro-inflammatory cytokines, interlukin-8 (IL-8) and interlukin-1 receptor antagonist (IL-1RA) levels in HaCaT cells were measured via commercially available ELISA kits (*R&D Systems, Inc.,* MN, USA). HaCaT cells were grown to confluency and seeded into 6-well cell culture plates (*Sarstedt*, Leicester, UK) at a density of 3 × 10^5^ cells per well for 24 h. Subsequently, the cells were serum starved for 24 h and treated with complete medium supplemented with either 1% (v/v) HPLC-grade methanol (V. ctrl) (*Merck*, Gillingham, UK); 25 μg mL^−1^ of LPS from *E. coli* (*Merck*, Gillingham, UK) (positive control for the assay); 20 μg mL^−1^ of each glycolipid congener; or SLES for a further 24 h (surfactants’ concentrations previously shown to have no inhibitory effects on the viability of HaCaT cells). Cell-free supernatant samples were generated for ELISA assessment as described in the previous paragraph and analysed with ELISA kits utilised as per the manufacturer’s instructions.

### Evaluation of immunomodulatory effects of glycolipids

Assessment of potential immunomodulatory effects of surfactants was carried out using the method described by Di Caprio et al. (2015) with slight modifications. HaCaT cells were cultured to confluency, seeded into 6-well cell culture plates (*Sarstedt*, Leicester, UK) at a density of 2 × 10^5^ cells per well, cultured for 24 h, and then serum starved for another 24 h. Thereafter, the cells were stimulated with 25 μg mL^−1^ LPS from *E. coli* (*Merck*, Gillingham, UK) for 24 h. Following LPS stimulation, the medium was aspirated and the cells were treated with complete medium supplemented with 1% (v/v) HPLC-grade methanol (V. ctrl) (*Merck*, Gillingham, UK) or 20 μg mL^−1^ of each glycolipid congener or SLES for a further 24 h. Cell-free supernatant samples were generated as described in the previous sub-section “Assessment of pro-inflammatory cytokine production”, and the protein levels of IL-8 and IL-1RA were measured using commercially available ELISA kits (*R&D Systems, Inc.,* MN, USA) utilised as per the manufactures instructions (Di Caprio et al. 2015).

### RNA extraction and cDNA synthesis

Total RNA was extracted from monolayer of HaCaT cells using TRIzol™ Reagent (*Invitrogen*, Paisley, UK), analysed integrity via agarose gel electrophoresis, and quantified using NanoDrop ND-1000 (*ThermoFisher Scientific*, Loughborough, UK). Total RNA extracts were reverse transcribed to generate cDNA samples using a G-STORM GS1 thermal cycler (*Gene Technologies Ltd*., Somerset, UK). Unless otherwise stated, all reagents for cDNA synthesis were sourced from *ThermoFisher Scientific*, Loughborough, UK. cDNA was synthesised in the following reaction mixture of 20 μL: 50 ng of total RNA, 12 μL nuclease-free double distilled water, 25 ng of Oligo(dT)_12–18_ primer, 10 mM DTT, 0.5 mM dNTP, and 10 U of SuperScript™ Reverse Transcriptase II (RT). The reaction mixture was incubated for 10 min at 70 °C to denature RNA, 2 min at 42 °C for primer hybridisation, 50 min at 42 °C for cDNA synthesis, and, finally, RT deactivation step at 70 °C for 15 min. Reverse transcriptase minus (NRT) negative control and no template negative control (NTC) were generated by supplementation of the respective components with molecular grade water (UltraPure™ distilled water) (*ThermoFisher Scientific*, Loughborough, UK).

### Quantitative real-time PCR (qPCR)

qPCR experiments were performed as per the Minimum Information for Publication of Quantitative Real-Time PCR Experiments (MIQE) guidelines (Bustin et al. 2009). qPCR was carried out in LightCycler480 II 96 multi-well plates (*Roche Diagnostics*, Burghess Hill, UK) using a LightCycler480 II (*Roche Diagnostics*, Burghess Hill, UK). Reactions were set up following the manufacturer’s instructions using SYBR® Green I master mix (*Roche Diagnostics*, Burghess Hill, UK), and the primer sets listed in Table S1. The qPCR cycling conditions were set at 95 °C for 5 min and 45 cycles for 30 s at 95 °C, 20 s at 60 °C, and 30 s at 72 °C. Three technical replicates of all experimental samples were analysed, and data were reported as fold change normalised to the house-keeping gene (*GAPDH*) relative to untreated control and computed as 2^−ΔΔCq^ (Maussion et al. 2021).

### Statistical analysis

Statistical analyses of all data were carried out using Prism v 9.4.1 (458) for MacOS (*GraphPad Software*, San Diego, CA, USA). Cell viability and AO/PI staining data was analysed via a two-way analysis of variance (ANOVA) followed by post hoc multiple comparison testing. ELISA and RT-qPCR data were analysed using a one-way ANOVA followed by post hoc multiple comparison testing. The significance of all results was tested at a level of *p* ≤ 0.05. LD_50_ was determined from three independent cell viability assays and reported as the mean and standard error from the mean. Significant differences in LD_50_ of each glycolipid congener in comparison to SLES was established by carrying out unpaired *t* tests at a level of *p* ≤ 0.05.

## Results

### Chemical characterisation and analysis of glycolipids’ surface activity

The relative percentage abundance and congener profile of all glycolipids utilised in this study were analysed via HPLC–MS/ESI and are fully detailed in a previous study (Adu et al. 2022). HPLC–MS/ESI analysis revealed that acidic SL was 100% pure and lactonic SL was 90% pure. The predominant congeners present in acidic SL and lactonic SL were acidic SL C18:1 (65.53%) and lactonic SL R1 + R2 = Ac, C18:1 (63.40%), respectively. For rhamnolipids, the mono-RL preparations were 96% pure and the di-RL 97% were pure. The most abundant congeners present in the mono-RL and di-RL were Rha-C_10_-C_10_ (84.40%) and Rha-Rha-C_10_-C_10_ (57.99%), respectively (Adu et al. 2022).

A comparative analysis of the surface activity of each glycolipid congener against SLES revealed that all glycolipid congeners utilised in this study had lower CMC values (0.03–0.06 mg mL^−1^) compared with SLES (0.66 mg mL^−1^). The di-RL preparation exhibited the greatest surface activity reducing the surface tension of water from 72 to 28.70 mN M^−1^ (Table S2). It is important, however, to mention that the glycolipids utilised in this study are not similar in chemical structures and composition with SLES and may account for the differences in their chemical properties and consequently their bioactivities (Fig. S1).

### Comparative effects of microbial glycolipid and SLES treatments on the viability of human keratinocytes

**Fig. 1:**
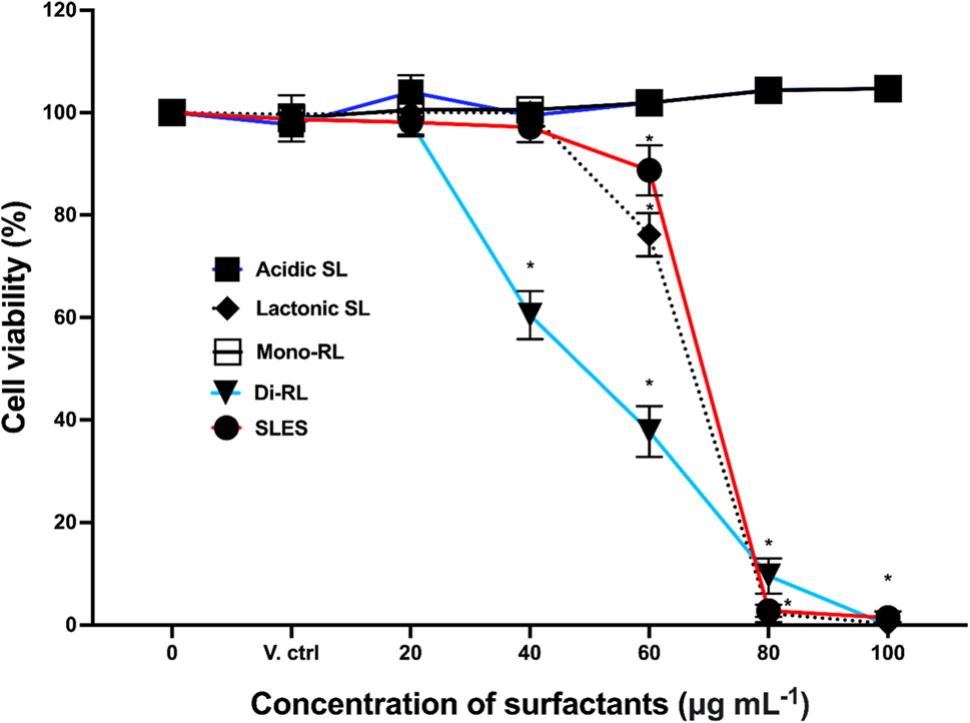
Microbial glycolipid congeners and SLES affect the viability human keratinocytes in a differential manner. HaCaT cells were treated with either 1% (v/v) methanol (V. ctrl) or 0–100 μg mL^−^.^1^ of acidic SL, lactonic SL, mono-RL, di-RL, and SLES. Data are the mean results of three independent experiments; error bars represent standard error from the mean. Statistical significance was determined using a one-way ANOVA followed by Dunnett’s multiple comparison test (*p ≤ 0.05)

The cytotoxicity effects of each highly purified glycolipid congener in comparison with SLES on the HaCaT cell line were assessed in vitro using an XTT cell viability assay. Viability of HaCaT cells post treatment with glycolipids, methanol, and SLES was expressed in percentage relative to the untreated control group. As expected, the vehicle control of 1% (v/v) methanol had no significant effects on HaCaT cells. Di-RL and lactonic SL significantly reduced the viability of HaCaT cells at concentrations above 40 and 60 µg mL^−1^, respectively (*p* < 0.0001, Fig. 1). Both acidic SL and mono-RL had no inhibitory effects on the viability of HaCaT cells up to 100 µg mL^−1^ (Fig. 1). When treatment concentration of acidic SL and mono-RL was increased up to 500 µg mL^−1^, mono-RL significantly reduced cell viability at concentrations exceeding 400 µg mL^−1^ (*p* < 0.0006, Fig. S2) while acidic SL demonstrated no inhibitory effects on HaCaT cells at concentrations as high as 500 µg mL^−1^ (Fig. S2). SLES significantly reduced the viability of HaCaT cells at concentrations above 60 µg mL^−1^ (*p* < 0.0096, Fig. 1). Comparing SLES with glycolipids, we observed significantly less viable cells in di-RL and lactonic SL treatment groups than in SLES only at 40 µg mL^−1^ and 60 µg mL^−1^, respectively (*p* > 0.0051). However, above 60 µg mL^−1^, the percentage of viable cells treated with SLES was significantly lower than cells treated with acidic SL and mono-RL (*p* ≤ 0.0028). These observations were further investigated by calculating the LD_50_ of each glycolipid congener and comparing with the calculated LD_50_ of SLES (Table 1). The LD_50_ for mono-RL was significantly higher than that of SLES (628.27 ± 47.61, 65.50 ± 1.26, respectively). As acid-SL had no discernible effect on cell viability up to the maximum concentration tested, the LD_50_ could not be calculated; it is, therefore, reasonable to assume that it is also significantly higher than that of SLES. Conversely, di-RL and lactonic SL were found to possess a lower LD_50_ (47.57 ± 2.76, 62.62 ± 1.33, respectively) than SLES; however, this was only significant for the di-RL.

**Table 1:** The LD_50_ of each glycolipid congener and SLES on HaCaT cells. The results are the mean values (± SEM) calculated from three independent cell viability assays. ND, not determined; Est, estimated LD_50_ concentration. (*Statistically significant result compared to SLES, *p* =  < 0.05)

**Surfactants**	**Mean LD**_**50**_** (**± **SEM)**
Acidic SL Lactonic SL Mono-RL Di-RL SLES	ND 62.62 μg mL^−1^ (± 1.33) 628.27 μg mL^−1^ (± 47.61)* ^(Est)^ 47.57 μg mL^−1^ (± 2.76)* 65.50 μg mL^−1^ (± 1.26)

### Comparative effects of microbial glycolipid and SLES treatment on the cell morphology of human keratinocytes

**Fig. 2:**
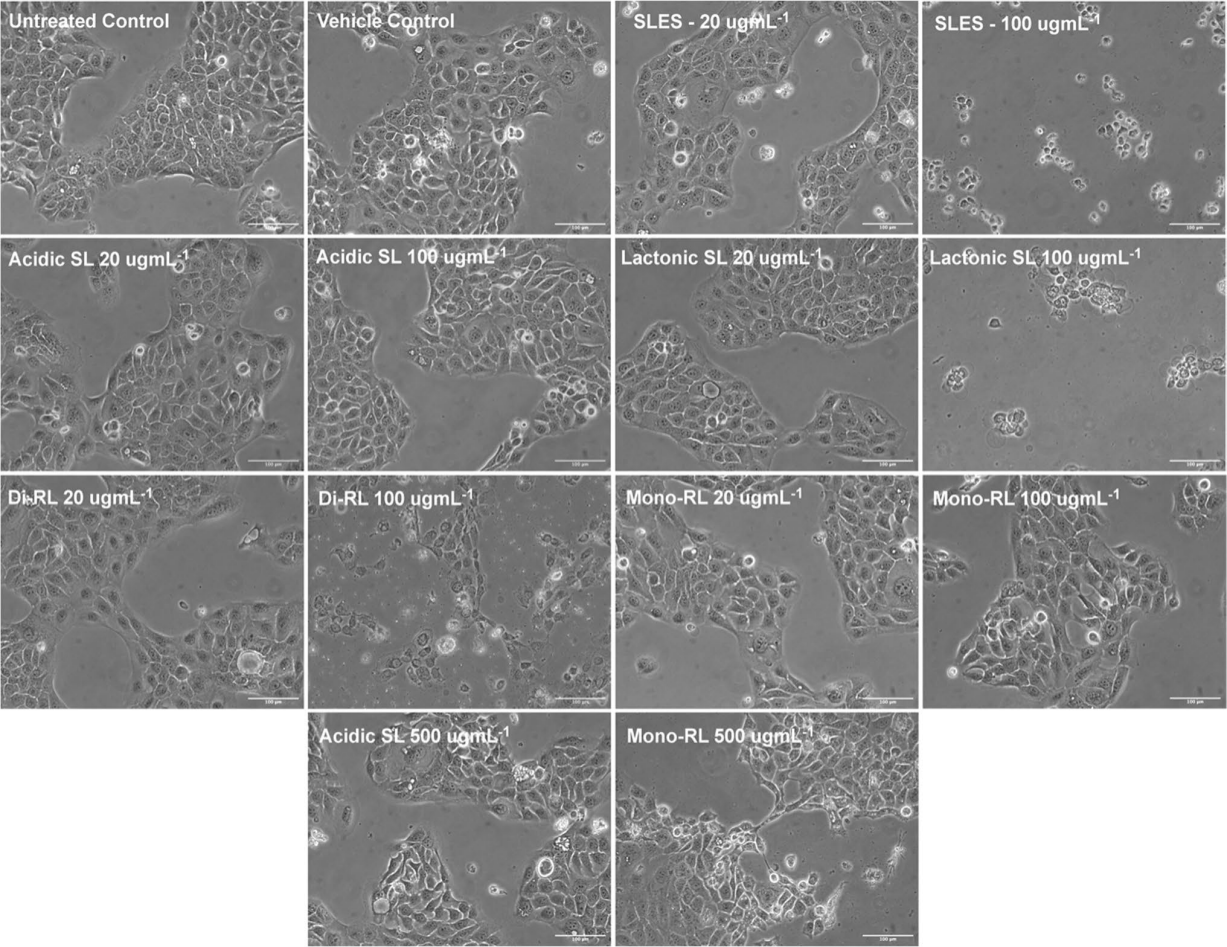
The effects of glycolipids and SLES on HaCaT cellular morphology and cellular detachment. Cells were directly observed at 200 × magnification following treatment with either 1% (v/v) methanol (V. ctrl), each microbial glycolipid congener or SLES at both 20 and 100 μg mL^−1^ for 24 h and compared to untreated controls. In the case of both acidic SL and mono-RL, cell morphology was also observed following treatment with up to 500 μg mL^−1^ for 24 h. Cell morphological observations were carried out independently three times with three replicates per treatment group. Each replicate was imaged in three independent locations within the well, and a representative image for publication was selected at random. Scale bar was set at 100 μm

HaCaT cells were treated for 24 h with 1% (v/v) methanol (V. ctrl), preparations of each glycolipid congener and SLES at concentrations of 20 μg mL^−1^ and 100 μg mL^−1^, and in the case of acidic SL and mono-RL 500 μg mL^−1^. Visible light microscopy was used to observe and compare changes in cell morphology to untreated cells. As expected, HaCaT cells in untreated and vehicle control groups remained adherent to the bottom of plates maintaining the normal flat cuboidal shape of keratinocytes (Fig. 2). Similarly, treatment with preparations of all glycolipid congeners and SLES at 20 μg mL^−1^ had no observable effect on HaCaT cellular morphology. However, treatment with lactonic SL, di-RL, and SLES at 100 μg mL^−1^ resulted in drastic reductions in cell population with the few adherent cells acquiring round/shrinking cell morphology (Fig. 2). Morphological changes in comparison to the untreated and vehicle control-treated cells were absent in cells treated with acidic SL at concentration as high as 500 μg mL^−1^, and in the cells treated with mono-RL, observable morphological changes were observed at 500 μg mL^−1^ (Fig. 2).

### Comparison of the mechanism of HaCaT cell death resulting from treatment with either microbial glycolipids or SLES

AO/PI dual staining technique was used to assess the morphological pattern of cell death in HaCaT cells following exposure to each microbial glycolipid congener and SLES for 24 h. AO is membrane permeable and stains live cells green with a non-fragmented intact nuclei appearance. Observations of non-intact, green-stained cells showing membrane blebbing and chromatin condensation are indicative of apoptotic cell death. PI is membrane impermeable and will only stain cells whose membrane integrity has been compromised as red or orange. The observation of cells staining red/orange with non-fragmented nuclei is indicative of necrotic cell death (Cummings and Schnellmann 2004; Atale et al. 2014). No morphological indications of apoptotic cell death were observed in any of the treatment conditions (Fig. S3).

**Fig. 3:**
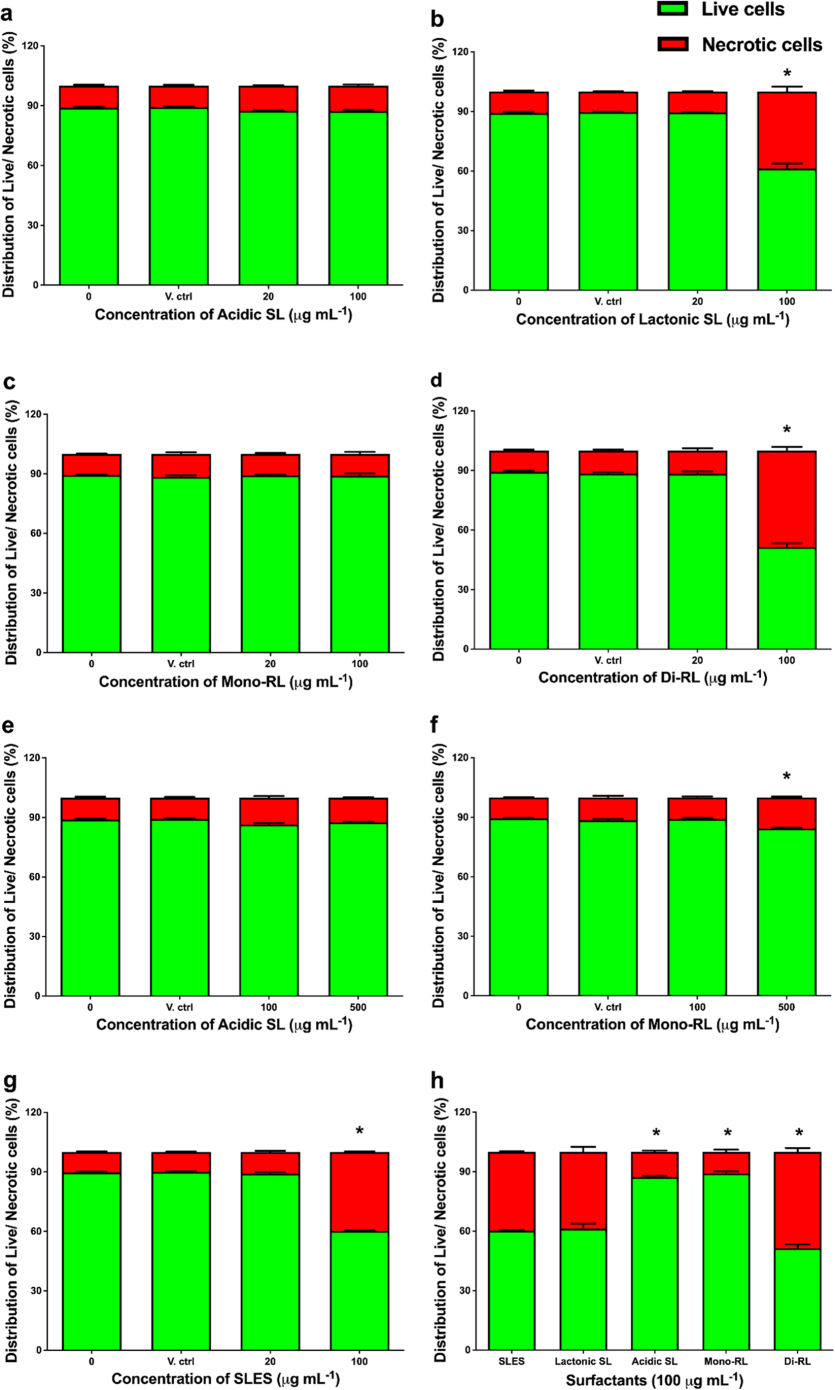
The use of AO/PI dual staining technique to assess the morphological pattern of cell death induced in HaCaT cells following treatment with each microbial glycolipid congener or SLES. Percentage of live cells (green) and necrotic cells (red) following treatment with 0, 20, or 100 μg mL^−1^ of acidic SL (**a**); lactonic SL (**b**); mono-RL (**c**); di-RL (**d**); 0, 100, or 500 μg mL^−1^ acidic SL (**e**); mono-RL (**f**); and 0, 20, or 100 μg mL^−1^ SLES (**g**); percentage live cells (green) to necrotic cells (red) following 100 μg mL^−^.^1^ treatment of each surfactant (**h**). Live/dead staining experiments were carried out independently three times, and three images per well were randomly selected and processed with ImageJ software for integrated density measurement. Statistical significance compared to untreated controls was determined using a two-way ANOVA followed by Šidák’s multiple comparison test. *p ≤ 0.05

HaCaT cells stained with either AO (live cells) or PI (necrotic cells) were imaged, and pixel brightness of the fluorescent images was measured as integrated density using ImageJ software. In comparison to untreated cells, there was no significant decrease in the percentage of live cells or increase in the percentage of necrotic cells following treatment with either the vehicle control or 20 μg mL^−1^ of all surfactant preparations (Fig. 3a–g). There were, however, significant decreases in the percentage of live cells and increases in the percentage of necrotic cells following treatment with lactonic SL, di-RL, and SLES at 100 μg mL^−1^ (*p* < 0.0001, Fig. 3b, d, g). Treatment with acidic SL and mono-RL at 100 μg mL^−1^ resulted in no significant difference in the percentage of live or necrotic cells (Fig. 3a, c). Additionally, further increasing the treatment concentration of acidic SL up to 500 μg mL^−1^ resulted in no significant difference in the percentage of live or necrotic cells (Fig. 3e). However, cells treated with mono-RL at 500 μg mL^−1^ did result in significant decrease in the percentage of live cells and increase in the percentage of necrotic cells (*p* < 0.0001, Fig. 3f). When directly comparing the percentage of live cells present following treatment with each preparation of glycolipid congener to SLES, no significant differences were observed at 20 μg mL^−1^ (Fig. 3h). At 100 μg mL^−1^, there was a significant increase in the percentage of live cells following treatment with either acidic SL or mono-RL (*p* < 0.0001, Fig. 3h) in comparison to SLES. Treatment with 100 μg mL^−1^ of lactonic SL resulted in no significant difference in the percentage of live cells when compared with treatment with 100 μg mL^−1^ SLES; however, treatment with di-RL resulted in a significant decrease in the percentage of live cells (*p* < 0.0001, Fig. 3h).

### Effect on the production of pro-inflammatory cytokines and expression of pro-inflammatory cytokine genes in human keratinocytes in response to treatment with glycolipids or SLES

**Fig. 4:**
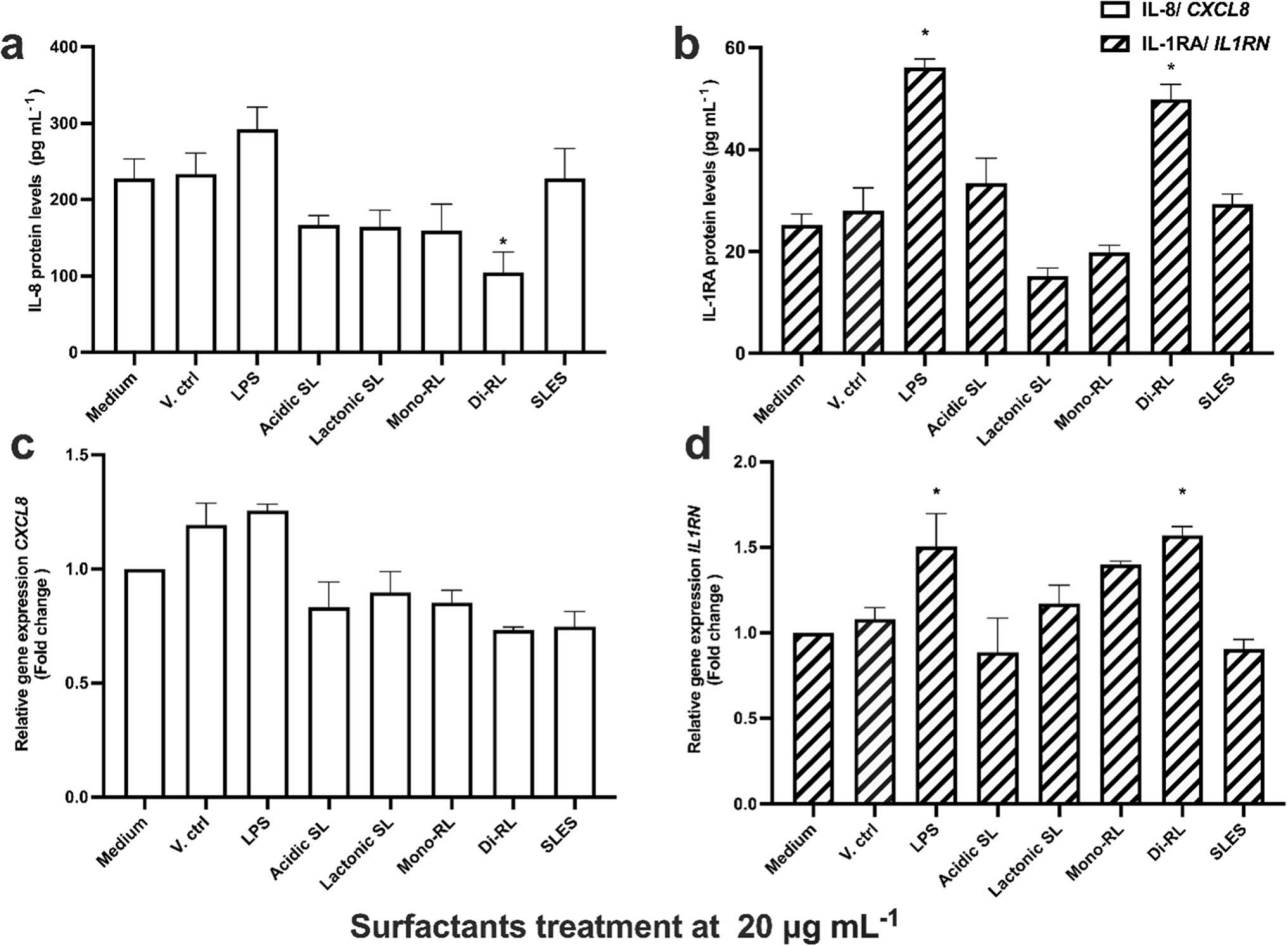
ELISA analysis of **a** IL-8 and **b** IL-1RA protein levels and RT-qPCR analysis of *CXCL8* (**c**) and **d** *IL1RN* expression in HaCaT cells. The cells were treated with complete medium (Medium), 1% (v/v) methanol (V. ctrl), LPS (25 μg mL^−1^), glycolipid preparations, and SLES (20 μg mL^−^.^1^) for 24 h. Data are the mean results of four independent experiments; error bars represent standard error from the mean. Statistical significance was determined using a one-way ANOVA followed by Dunnett’s multiple comparison test. *p ≤ 0.05

The effect on the production of pro-inflammatory cytokines and the expression of their respective genes in HaCaT cells following treatment with each glycolipid congener and SLES were investigated using both immunological assays and reverse transcription qPCR (RT-qPCR). Initial screening of pro-inflammatory cytokine production using a semi-quantitative array revealed that treatment with lactonic SL and di-RL stimulate the production of IL-8 at threshold level sufficient for detection via ELISA (Fig. S4). Using this result as a guide to further investigation, the effect on IL-8 and IL-1RA cytokine production in HaCaT cells treated with each glycolipid congener in comparison to treatment with SLES was assessed by ELISA. Treatment with 1% (v/v) methanol (V. ctrl), 20 μg mL^−1^ each of acidic SL, lactonic SL, and mono-RL, and SLES preparations had no significant effect on IL-8 and IL-1RA production in HaCaT cells (Fig. 4a, b). However, di-RL significantly attenuated IL-8 protein levels (*p* = 0.0271) while significantly inducing IL-1RA production (*p* = 0.0031) (Fig. 4a, b). More importantly, in comparing cytokine production levels in HaCaT cells treated with each glycolipid congener with SLES, we observed significantly higher levels of IL-1RA (*p* = 0.0011) in di-RL-treated cells (Fig. 4b). Although IL-8 level in di-RL was reduced in comparison to SLES, this was not statistically significant (Fig. 4a).

The expression of *CXCL8* and *IL1RN* genes was assessed in HaCaT cells treated with both microbial glycolipids and SLES preparations at 20 μg mL^−1^ using RT-qPCR. Similar to the results from the ELISAs, no significant change in the expression of either gene was observed following treatment of HaCaT cells with SLES or each glycolipid congener except for di-RL (Fig. 4c, d). When treated with di-RL, a trend toward decreased expression of *CXCL8* was observed and expression of *IL1RN* was significantly increased when compared to untreated cells (*p* = 0.0117, Fig. 4c, d). Interestingly, when comparing cells treated with each glycolipid congener and those treated with SLES, a significant increase in *IL1RN* expression was observed in di-RL (Fig. 4d). However, no significant difference in the expression of *CXCL8* was observed when comparing HaCaT cells treated with each glycolipid congener and SLES (Fig. 4c).

### Immunomodulatory effects of glycolipids and SLES in LPS-stimulated human keratinocytes

**Fig. 5:**
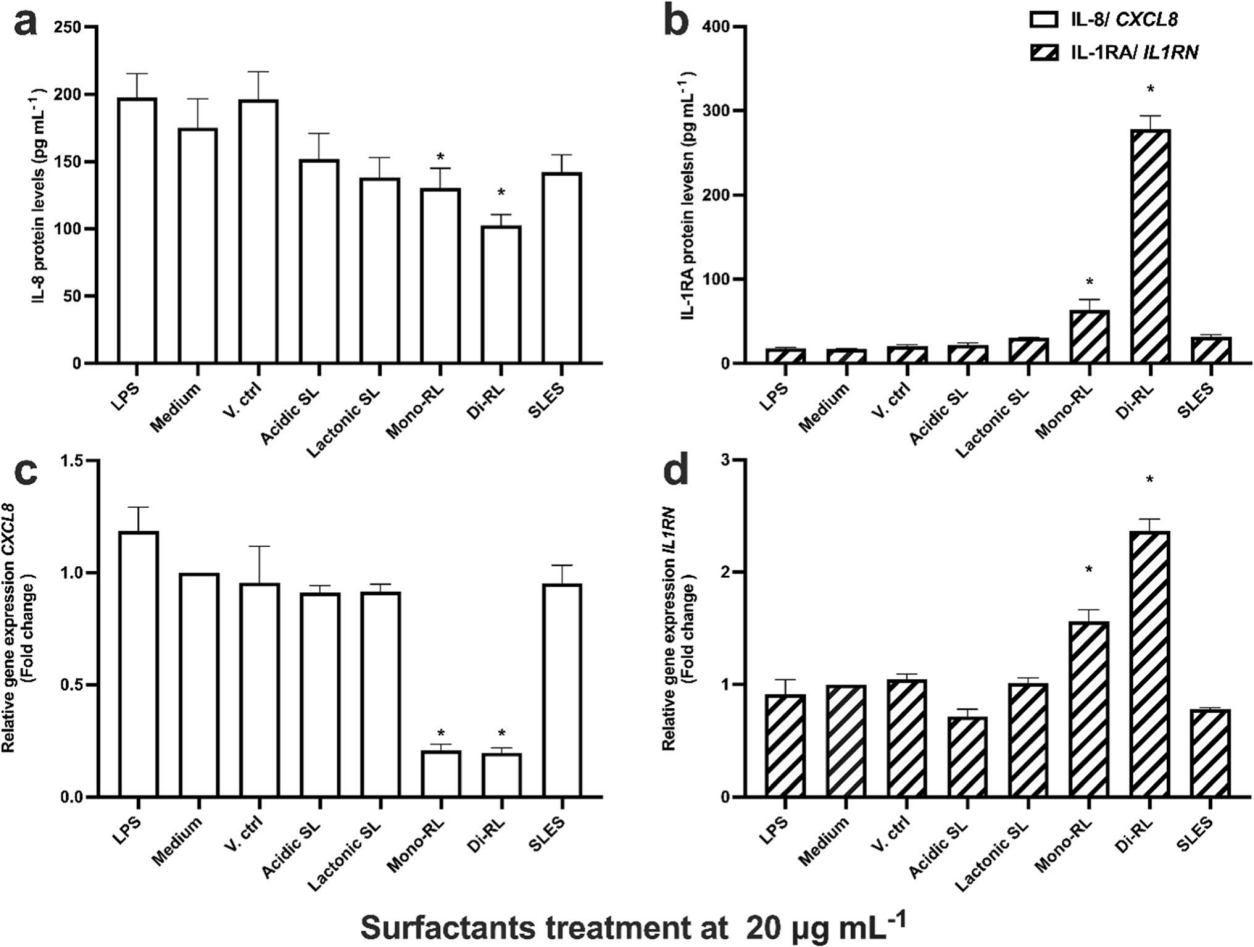
ELISA analysis of **a** IL-8 and **b** IL-1RA protein levels and RT-qPCR analysis of *CXCL8* (**c**) and **d** *IL1RN* expression in HaCaT cells. The cells were pre-treated with 25 μg mL^−1^ LPS for 24 h and thereafter cultured in cultured in complete medium (Medium), complete medium supplemented with 1% (v/v) methanol (V. ctrl), or glycolipid preparations and SLES at 20 μg mL^−^.^1^ for 24 h. Data are the mean results of four independent experiments; error bars represent standard error from the mean. Statistical significance was determined using a one-way ANOVA followed by Dunnett’s multiple comparison test. *p ≤ 0.05

HaCaT cells were pre-treated with LPS at 25 μg mL^−1^ to simulate an inflammatory response such as would occur in psoriasis infections and afterwards treated with each glycolipid congener and SLES preparations at 20 μg mL^−1^ to investigate their potential ameliorative/immunomodulatory effects. Following both stimulation and treatment, IL-8 and IL-1RA protein levels and *CXCL8* and *IL1RN* expression levels in HaCaT cells were measured by ELISA and RT-qPCR, respectively. Treatment with 1% (v/v) methanol had no significant effect on cytokine production in HaCaT cells following stimulation with LPS. SLES, acidic SL, and lactonic SL had no significant effect on IL-8 and IL-1RA production in HaCaT cells stimulated by LPS (Fig. 5a, b). Interestingly, treatment with both di-RL and mono-RL significantly attenuated IL-8 protein levels in HaCaT cells (*p* = 0.0028 and *p* = 0.0456, respectively) while increasing IL-1RA protein levels in HaCaT cells (*p* < 0.0001 and *p* = 0.0009, respectively) following stimulation with LPS (Fig. 5a, b). Consistent with these results, *CXCL8* gene expression was significantly decreased in HaCaT cells stimulated with LPS and then treated with either di-RL or mono-RL (*p* < 0.0001). Additionally, the expression of *IL1RN* was increased in HaCaT cells stimulated with LPS and then treated with either di-RL or mono-RL (*p* < 0.0001and *p* = 0.0002, respectively) (Fig. 5c, d). Significant decreases in *CXCL8* and increases in *IL1RN* gene expression were also observed in mono and di-RL treatment groups when comparing LPS-stimulated HaCaT cells treated with glycolipids with SLES (*p* < 0.0001).

## Discussion

The growing consumer concerns over skin irritations and allergic reactions arising from the use of synthetic ingredients in skincare formulations have expanded research in the cosmeceutical and biotechnology industries to replace these ingredients with natural, biocompatible, and sustainable alternatives (Seweryn 2018). In this study, we assessed the cytotoxicity and immunomodulatory effects of different microbially derived glycolipid congeners on human keratinocytes in comparison with SLES, a synthetic surfactant commonly utilised in skincare formulations. In general, this study demonstrated that highly purified glycolipid congeners have differing effects on human keratinocytes. Also, at high concentrations, acidic SL and mono-RL have negligible cytotoxicity effects on human keratinocytes compared with SLES; at non-inhibitory concentrations, mono-RL and di-RL modulate cytokines produced in LPS-stimulated human keratinocytes.

There is a significant body of research investigating the effects of glycolipids on various mammalian cell types either in a diseased or “normal” state, but only a few of these studies have so far focused on healthy human skin cells (Lydon et al. 2017; Maeng et al. 2018; Voulgaridou et al. 2021). The cytotoxicity effects of rhamnolipids extracted from *Pseudomonas* strain MCTG214(3b1) (mainly comprising of di-RL) and *Marinobacter* strain MCTG107b (composed of di-RL and mono-RL mixtures) demonstrated in vitro against HaCaT cells and transformed liver epithelial cells (THLE3) (Voulgaridou et al. 2021). The authors reported that up to 0.25 mg mL^−1^ treatment concentrations, the rhamnolipids exhibited negligible cytotoxicity effects against both cell lines whereas the synthetic surfactants induced cytotoxicity effects at treatment concentrations as low as 0.002 mg mL^−1^ (Voulgaridou et al. 2021). In another study, Maeng et al. (2018) demonstrated that sophorolipid mixtures (comprising of lactonic and acidic forms) synthesised from hydrolysed horse oil exhibited cytotoxicity effects on skin fibroblast cells only at concentrations above 50 µg mL^−1^. Low concentrations of these sophorolipids improved wound healing (0.5–5 µg mL^−1^) and attenuated pro-inflammatory cytokines produced in LPS-stimulated macrophages (5–25 µg mL^−1^) (Maeng et al. 2018). From the above reports, although significant bioactive properties of glycolipids were investigated using healthy human skin cells, the authors either utilised only a single class of impure glycolipids or glycolipid preparations having a mixture of various congeners. Hence, the bioactivities observed could not be assigned to a specific congener. Here, a broader range of glycolipid congeners highly purified and properly characterised were utilised. HPLC–MS/ESI analysis showed that acidic SL was 100% pure and lactonic SL was 90% pure. Also, mono-RL preparations were 96% pure and the di-RL were 97% pure (Adu et al. 2022). This high level of the glycolipids’ purity was sufficient to attribute the effects observed in human keratinocytes as being caused by the individual glycolipid congeners. Moreover, measurement of surface activity revealed lower CMC values in all glycolipid congeners (0.03–0.06 mg mL^−1^) than SLES (0.66 mg mL^−1^). Consequently, in skincare applications, lower amounts of these glycolipids would be required to form micelles and to perform surface activities such as foaming and emulsification (Rahimi et al. 2019; Perinelli et al. 2020).

Cytotoxicity is characterised by adverse effects on cells caused by treatment agents post exposure at known concentration within a specified time; hence, methods for assessing cytotoxicity effects require that effects of the treatment agents on cellular functions and integrity be compared to untreated cells and the effects measured within a specified time (Cummings and Schnellmann 2004; Leoty-Okombi et al. 2021). The concentration of SLES utilised in most skincare products is at a dose rage of 0.01 to 50% (v/v), and for toxicological studies, the organisation for Economic Cooperation and Development (OECD; test no. 439) recommends the use of up 5% (v/v) sodium dodecyl sulphate (SDS) as positive control (Leoty-Okombi et al. 2021; OECD 2021). Keeping to these standards, XTT cell viability assay was utilised to comprehensively assess the effects of highly purified sophorolipid and rhamnolipid congeners on the viability of human keratinocytes in comparison with SLES at concentrations ranging from 0 to 100 µg mL^−1^ for 24 h. For acidic SL and mono-RL, further experiments increasing treatment concentrations up 500 μg mL^−1^ were performed. This range of concentrations utilised was sufficient to produce dose response in the human keratinocytes. In summary, we demonstrated that microbial glycolipids have differing effects on human keratinocytes dependent on their chemical structure and that acidic SL and mono-RL have negligible cytotoxicity effects in comparison with SLES; while SLES were significantly inhibiting HaCaT cells at concentrations above 60 µg mL^−1^, no cytotoxicity effects were observed in mono-RL and acidic SL at up to 300 μg mL^−1^ and 500 μg mL^−1^, respectively. Effects of the surfactants on HaCaT cell viability were further investigated via LD_50_ analysis. Here, except for acidic SL whose LD_50_ could not be determined, the LD_50_ of mono-RL was the highest among all other surfactants. LD_50_ is the amount of substance (drug) required to cause the death of 50% of cells or an organism within a specified time (Adamson 2016). In toxicological studies, LD_50_ analysis is critical for drug safety evaluation and standardisation in that the higher the LD_50_, the safer the drug (Zhang et al. 2022). Thus, the undetermined LD_50_ in acidic SL and the highest recorded LD_50_ in mono-RL-treated cells coupled with their high inhibitory concentrations compared with SLES suggest that these glycolipids have less cytotoxicity effects on human keratinocytes and could potentially offer a suitable substitute to SLES.

Although currently there are not enough studies to fully understand the mechanisms by which glycolipids may or may not affect skin cells, it is worthy of note that the differing cytotoxicity effects of the various glycolipid congeners demonstrated in this study could be attributed to factors such as their hydrophilicity/hydrophobicity, biological origin, surface tension reduction ability, congener profile, their chemical structure and properties, and intercellular and intracellular organisation of the keratinocytes utilised (Rahimi et al. 2019). One notable study that explored these potential mechanisms examined the cytotoxicity effects of mono-RL and di-RL on MCF-7 human breast cancer cells (Rahimi et al. 2019). Considering that the less amount of sugar heads in mono-RL make them more hydrophobic than di-RL, the authors hypothesised that there was stronger interaction between the more hydrophobic surfaces of the MCF-7 human breast cancer cells, thereby ensuring greater impact on cell viability (IC_50_ = 25.87 µg mL^−1^) than in di-RL-treated cells (IC_50_ = 31 µg mL^−1^) (Rahimi et al. 2019). Accordingly, in this study, in view of the fact that keratinocytes possess membrane-rich hydrophilic proteins and intracellular hydrophilic channels, their interaction with the more hydrophilic and highest surface-active Di-RL could account for their lowest LD_50_ among all the glycolipid congeners (Juurlink and Sivilotti 2007; Mundstock et al. 2015). Furthermore, the relatively strong interaction between the less anionic Di-RL and negatively charged functional groups on the membrane of the skin cells coupled with electrophilic properties of these glycolipids is an additional interactive mechanism worth considering (Shao et al. 2017). Notwithstanding, in the future, further mechanistic studies would be required to better understand the interaction of rhamnolipids with the human skin. With regards to sophorolipids, their effects on cell viability are hypothesised to be associated with their degree of acetylation and saturation of fatty acid groups (Shao et al. 2012; Callaghan et al. 2022). In a study on the bioactivity of ten sophorolipids differing in molecular structures against human oesophageal cancer cells, the authors demonstrated that diacetylated sophorolipids exhibited higher cytotoxicity effects (MIC = 30 µg mL^−1^) than monoacetylated groups (MIC = 60 µg mL^−1^) (Shao et al. 2012). Similarly, sophorolipid mixtures majorly comprising of diacetylated sophorolipids (40.12%) exhibited cytotoxicity effects on skin fibroblasts at concentrations above 50 µg mL^−1^ (Maeng et al. 2018). On the contrary, for acidic SL, irrespective of their level of acetylation, they were demonstrated to have minimal effects on human oesophageal cancer cells (Shao et al. 2012). In another study conducted by Lydon et al. (2017), nonacetylated acidic sophorolipids were shown to have no cytotoxicity effects on HaCaT cells at concentrations above 500 µg mL^−1^, which are all in agreement with this present study (Lydon et al. 2017).

In terms of the effects of glycolipids and SLES on cell morphology and the pattern of cell death-induced post exposure to the human keratinocytes, again we showed that the glycolipids have differing effects on HaCaT cells with significantly less necrosis-inductive effects than SLES. Specifically, while acidic SL and mono-RL were demonstrated to have negligible effects on both cell morphology and induction of necrotic cell death at concentrations above 500 µg mL^−1^, SLES drastically reduced cell population via induction of necrotic cell death at concentrations above 100 µg mL^−1^. Induction of necrosis in living cells post surfactant treatment is hypothesised to be associated with the membrane penetrative effects of the surfactants at certain concentrations. This results in alteration of the cell membrane potential, carbon chain arrangements, dehydration of cell bilipid layer, and ultimately cell death (Callaghan et al. 2016, 2022; Shao et al. 2017). Thus, glycolipids such as acidic SL and mono-RL with less penetrative effects and biophysical interactions with the human keratinocytes were reported to have minimal effects on necrosis induction even at high concentrations. These findings agree with the cell viability analysis and in summary suggest that the glycolipids utilised in this study have innocuous effects on human keratinocytes and could potentially offer a safer alternative to SLES in skincare applications.

The primary function of the human skin is to serve as a physical, chemical, and biological barrier to external body surfaces and internal organs through specialised and highly regularised immune cells (Nguyen and Soulika 2019; Yousef et al. 2022). Consequently, in the event the human skin is exposed to foreign agents such as skin pathogens and toxic chemicals, immune responses may be initiated to ensure tissue homeostasis and repair (Nguyen and Soulika 2019). Hence, to further investigate the safety of microbial glycolipids for potential skincare applications, we assessed the effects of all purified glycolipid congeners on the IL-8 and IL-1RA cytokine production and their associated gene expressed at non-inhibitory concentration in comparison with SLES. Except for di-RL, no other surfactant had significant effects on either cytokine production or gene expression levels in HaCaT cells. However, in the di-RL-treated cells, there was an inverse relationship between IL-8 and IL-1RA protein levels in that while there was significant reduction in IL-8 protein secretion, we recorded significant increases in IL-1RA protein levels. Generally, IL-8 is known for activating and recruiting neutrophils to sites of infection via the IL-1 and TNF-α signalling pathways (Russo et al. 2014; Matsushima et al. 2022). Conversely, IL-1RA is a naturally occurring anti-inflammatory cytokine and acts as competitor to the binding site of IL-1β (Herder and Donath 2015; Kaneko et al. 2019). The binding of IL-1RA to the binding site of IL-1β prevents the binding of IL-1β, thereby inhibiting pro-inflammatory cytokines that could have been otherwise initiated by IL-1β (Herder and Donath 2015). Therefore, the production of IL-1RA by di-RL may have altered the IL-1 pathway by inhibiting the activation of regulatory pro-inflammatory cytokine mediators such as mitogen-activated protein kinases (MAPKs), Ikappa kinase β (IKKβ), and nuclear factor-kappa β (NF-κβ), accounting for the inverse relationship between IL-8 and IL-1RA production (Sajid et al. 2020; Matsushima et al. 2022). However, as there are currently no mechanistic studies to these effects, further investigations would be necessary.

LPS is an inflammatory substance that induces the synthesis of nitric oxide and the expression of pro-inflammatory markers such as TNF-α and several interleukins after binding to toll-like receptor four (TLR-4) on cell surfaces resulting in downstream signalling transduction by NF-κB (Maeng et al. 2018; Sun et al. 2019). Here, we investigated whether post keratinocyte stimulation with LPS, glycolipids could ameliorate/modulate the cytokines produced. Using ELISAs and RT-qPCR, we demonstrated that of all surfactants tested at non-inhibitory concentrations, only mono-RL and di-RL significantly attenuated IL-8 production and *CXCL8* expression levels while increasing IL-1RA and *IL1RN* levels after LPS-stimulated HaCaT cells were treated with glycolipids and SLES for 24 h. Psoriasis is a common skin disease affecting 60 million people worldwide and 1.52% of people in the UK (Raharja et al. 2021). Psoriasis is characterised by hyperproliferation of keratinocytes and massive accumulation of inflammatory mediators such as neutrophils and cytokines (majorly IL-8) (Baliwag et al. 2015; Mylonas and Conrad 2018). Therefore, treatment methods for psoriasis targeted at modulating *CXCL8* expression such as demonstrated by mono-RL and di-RL in this study could be an important step towards psoriasis treatments (Russo et al. 2014). Moreover, the higher IL-1RA and *IL1RN* levels post keratinocyte stimulation with LPS suggest that mono-RL and di-RL may have therapeutic potential to induce anti-inflammatory mediators in diseased skin to modulate the continued cascade of pro-inflammatory cytokines that may have otherwise implicated an already establish skin infection (Herder and Donath 2015). Studies on immunomodulatory effects of glycolipids are quite rare. Moreover, most of these studies were performed using sophorolipids only. For instance, using immunoglobulin E (IgE) producing myeloma (U266 cells), sophorolipids extracted from *Candida bombicola* decreased IgE and gene expression levels of STAT3, TLR-2, and IL-6 (Hagler et al. 2007). Similarly, these sophorolipids decreased asthma severity in vivo by reducing Ova-specific IgE production in asthma-infected mouse model (Lee et al. 2008). More recently, sophorolipids synthesised from hydrolysed horse oil were demonstrated to reduce gene expression levels of TNF-α, COX-2, and IL-6 in mouse macrophages in a dose-dependent manner (5–25 µg mL^−1^) (Maeng et al. 2018). It must be noted that in our study, the sophorolipids utilised had no immunomodulatory effects. Nonetheless, the difference in the results reported could be attributed to the purity of the sophorolipids used, type of cells understudy, and difference in experimental design. However, the promising immunomodulatory effects of rhamnolipids demonstrated in this study would be worth exploring further.

Taken together, in this study, we have demonstrated that highly purified microbial glycolipids have differing effects on human keratinocytes depending on their chemical structure. Moreover, compared with SLES, acidic SL and mono-RL have negligible effects on keratinocyte viability, morphology, and production of pro-inflammatory cytokines. Furthermore, at non-inhibitory concentrations, di-RL and mono-RL modulate cytokine production and associated gene expression in LPS-stimulated human keratinocytes. These findings suggest that as potential innocuous and naturally derived surfactants, microbial glycolipids could potentially provide a safer and suitable alternative to SLES in skincare applications and, as an added functionality, perform immunopharmacological roles in topical skin infections such as psoriasis. This is the first time such a comprehensive study on glycolipid safety assessment and potential benefits to the human skin has been carried out. Notwithstanding, further in vitro studies including the use flow cytometry, reactive oxygen species induction, and comet assays to assess cellular DNA breakage coupled with robust mechanistic studies should be employed to further investigate the safety of glycolipids and their potential benefits to the human skin. Additionally, to accurately mimic the complex anatomy of the human skin, its physiological functions, and interactions with the human skin microbiome post glycolipid exposure, full thickness 3D in vitro skin models would be an important future step, bearing in mind to keep to the OECD standards for testing acute toxicity.

## Supplementary Information

Below is the link to the electronic supplementary material.

Supplementary file1 (PDF 776 KB)

## Data Availability

The datasets generated during and/or analysed during the current study are available from the corresponding authors on reasonable request.
