# Supplementary material for: Characterisation of cytotoxicity and immunomodulatory effects of glycolipid biosurfactants on human keratinocytes
Source: Appl Microbiol Biotechnol. 2022 Nov 28;107(1):137–52. doi: 10.1007/s00253-022-12302-5 (PMC9750927; doi:10.1007/s00253-022-12302-5)
Supplement: Supplementary file 1 — Supplementary file1 (PDF 776 KB) [file 253_2022_12302_MOESM1_ESM.pdf]

APPLIED MICROBIOLOGY AND BIOTECHNOLOGY

Supplementary material for:

Characterisation of cytotoxicity and immunomodulatory effects of glycolipid biosurfactants on human keratinocytes

Simms A. Adu [1], Matthew S. Twigg [1], Patrick J. Naughton [1], Roger Marchant [1] and Ibrahim M. Banat [1,] *

***Corresponding Author: Ibrahim M. Banat** im.banat@ulster.ac.uk
Tel.: +44 (0) 2870-123-062

[1]School of Biomedical Science, Ulster University, Coleraine BT52 1SA, UK.

ORCID
SAA: 0000-0002-7822-1634
MST: 0000-0002-8601-1960
PJN: 0000-0001-5995-9468
RM: 0000-0001-5213-229X
IMB: 0000-0001-8964-2840

Supplementary Figures

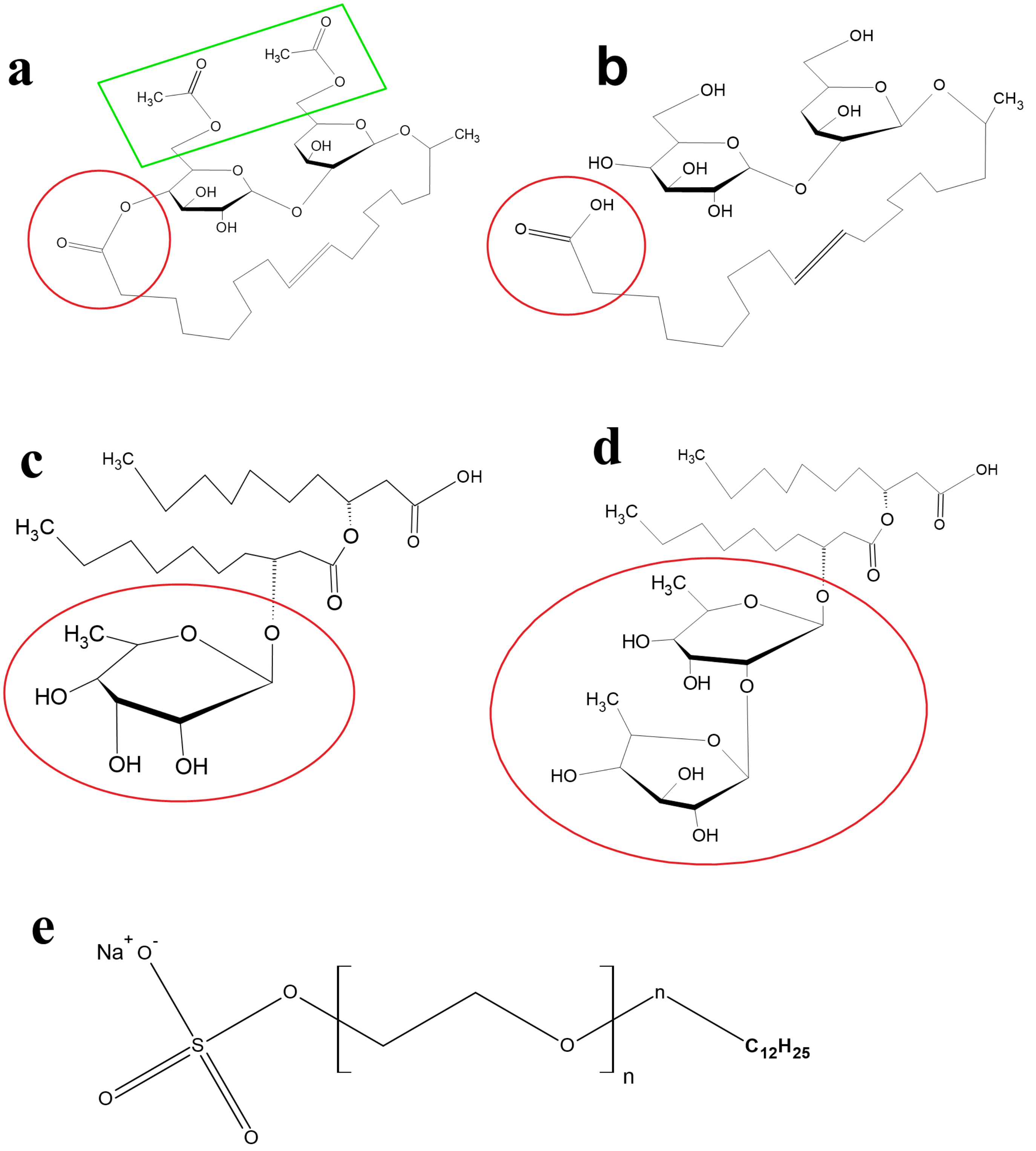

Fig. S1

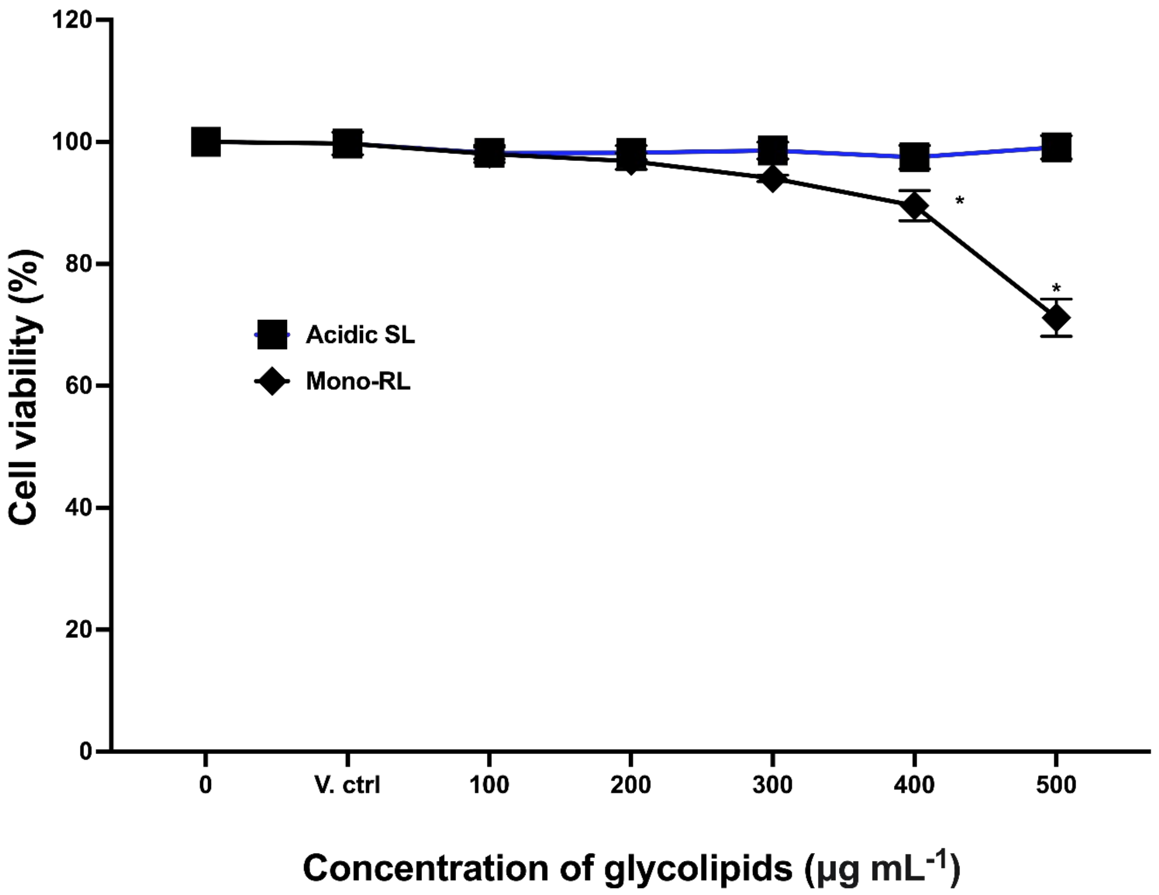

120
100
80
60
40
20
0
Cell viability (%)
Acidic SL
Mono-RL
*
*
0
V. ctrl
100
200
300
400
500
Concentration of glycolipids (μg mL-1)

Fig. S2

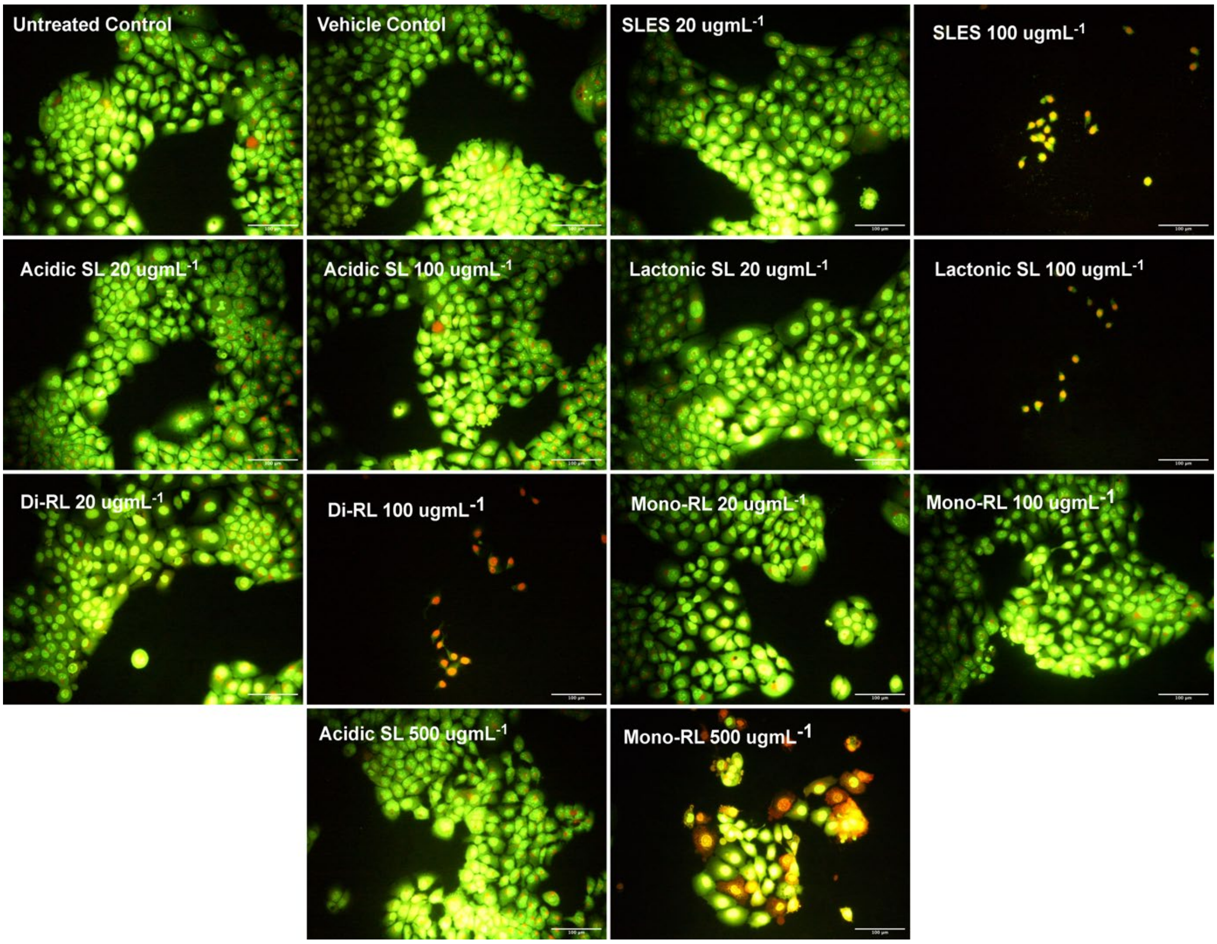
Untreated Control
Vehicle Contol
SLES 20 ugmL^{-1}
SLES 100 ugmL^{-1}
Acidic SL 20 ugmL^{-1}
Acidic SL 100 ugmL^{-1}
Lactonic SL 20 ugmL^{-1}
Lactonic SL 100 ugmL^{-1}
Di-RL 20 ugmL^{-1}
Di-RL 100 ugmL^{-1}
Mono-RL 20 ugmL^{-1}
Mono-RL 100 ugmL^{-1}
Acidic SL 500 ugmL^{-1}
Mono-RL 500 ugmL^{-1}

Fig. S3

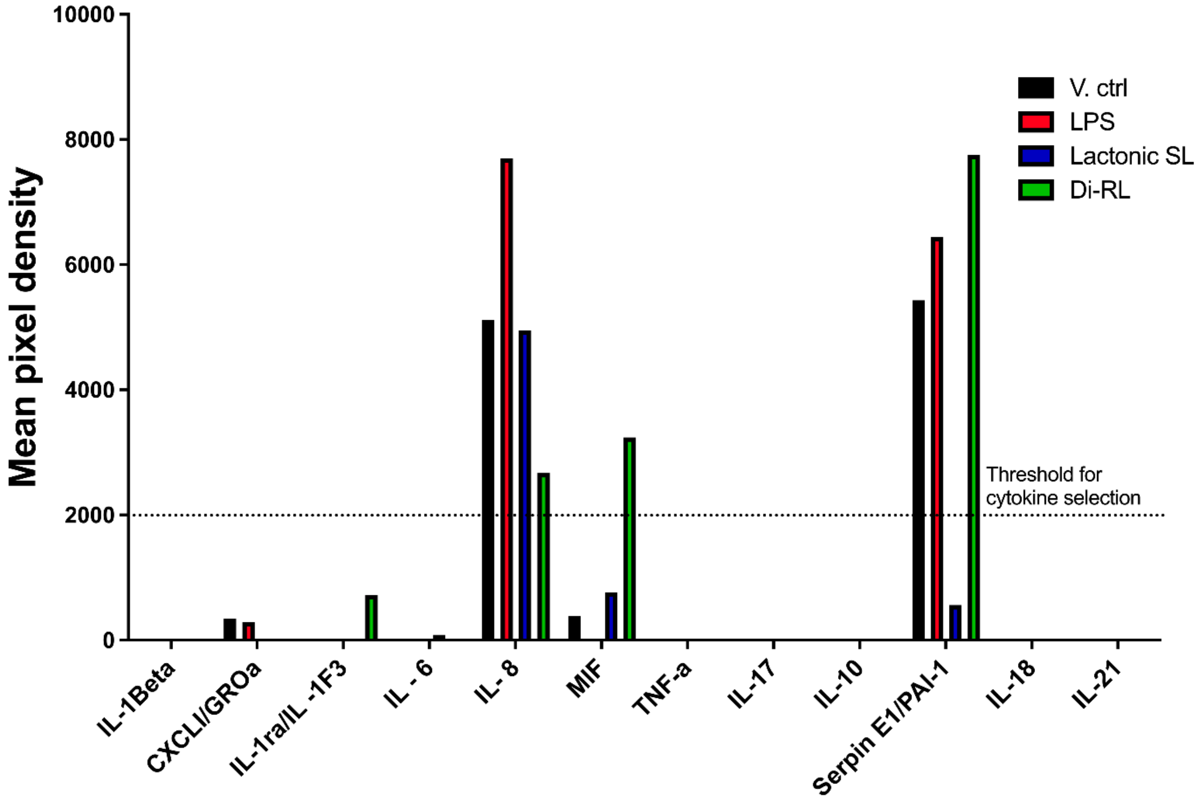

Mean pixel density
10000
8000
6000
4000
2000
0
V. ctrl
LPS
Lactonic SL
Di-RL
Threshold for cytokine selection
IL-1Beta
CXCLI/GROa
IL-1ra/IL -1F3
IL - 6
IL- 8
MIF
TNF-a
IL-17
IL-10
Serpin E1/PAI-1
IL-18
IL-21

Fig. S4

Supplementary figure legends

Fig. S1 Chemical structure of (a) Lactonic SL (di-acetylated with hydroxy fatty acid tail esterified at the 6' or 6" ends); (b) Acidic SL (non-acetylated with free fatty acid tail); (c) mono-RL (one rhamnose as hydrophilic moiety); (d) di-RL (two rhamnose as hydrophilic moiety) and (e) SLES. Figure adapted from Banat et al., 2010 and created with ChemSketch.com.

Fig. S2 The effects of Acidic SL and Mono-RL on the viability of human keratinocytes at treatment concentrations between 100 and 500 µg mL^{-1}. Data are the mean results of three independent experiments with each treatment group having six technical replicates, error bars represent standard error from the mean. Statistical significance was determined using a one-way ANOVA followed by Dunnett's Multiple comparison Test (* = $p \leq 0.05$).

Fig. S3 The use of AO/PI dual staining technique to assess the morphological pattern of cell death induced by surfactant treatment. Fluorescent microscopy images at 200 x magnification of untreated HaCaT cells (Untreated control); cells treated with 1% methanol (v/v) (Vehicle control); preparations of each glycolipid congener and SLES at 20 and 100 µg mL^{-1}. For Acidic SL and Mono-RL, treatment concentration was increased up to 500 µg mL^{-1}. Three images per well were randomly selected and processed with ImageJ Software for scale bar attachment ; scale bar was set at 100 µm.

Fig. S4 Semi-quantitative pro-inflammatory cytokine profile of cell-free supernatant samples from HaCaT cells treated with 1% methanol (v/v) (V. ctrl); 25 µg mL^{-1}LPS (positive control for the assay); or LD_{50} concentrations of Lactonic SL and Di-RL.

Supplementary tables

Table S1 Sequences of primer sets and product length of pro/ anti-inflammatory markers used for RT-qPCR analysis. Primers were designed using NCBI Primer-BLAST software and sequences of primers were compared to the NCBI nucleotide data bases via BLASTn Search tool.

**Primer**	**Gene**	**Sequence (5' – 3')**	**Amplicon Length (bp)**	**Annealing Temperature** (°C)
GAPDH SA-F	*GAPDH*	TCG ACA GTC AGC CGC ATC TT	80	49
GAPDH SA-R	*GAPDH*	CTC CGA CCT TCA CCT TCC CC	80	53
IL-8 SA-F	*CXCL8*	ACA CTG CGC CAA CAC AGA AA	117	47
IL-8 SA-R	*CXCL8*	TTC TCA GCC CTC TTC AAA AAC TTC	117	49
IL-RA SA-F	*IL1RN*	CCA AGA AGG CAA GAG CAA GCA	103	49
IL-RA SA-F	*IL1RN*	GCC CCA GCA GTT TAT GGG TT	103	49

Table S2 Critical micelle concentration (CMC) determination of microbial glycolipids and SLES. Surface activity of glycolipids and SLES were analysed using KRUSS K10 ST digital tensiometer. CMC was estimated from extrapolated intercepts of values plotted on both the X and Y -axes of XY graph. All surface tension measurements were taken in triplicate.

**Surfactants**	**CMC (mg mL $^{-1}$)**	**Surface tension (mN m^{-1})**
Acidic SL	0.06	43.00
Lactonic SL	0.06	35.37
Mono - RL	0.03	41.60
Di–RL	0.06	28.70
SLES	0.66	32.20
